# Accuracy of insulin resistance indices for metabolic syndrome: a cross-sectional study in adults

**DOI:** 10.1186/s13098-018-0365-y

**Published:** 2018-08-20

**Authors:** Luciana Pavan Antoniolli, Bárbara Limberger Nedel, Tassia Cividanes Pazinato, Leonardo de Andrade Mesquita, Fernando Gerchman

**Affiliations:** 1 Scientific Initiation Program, Faculty of Medicine, Federal University of Rio Grande do Sul, Rua Ramiro Barcelos, 2400, Second floor, Porto Alegre, RS 90035-003 Brazil; 2 Metabolism Unit, Endocrinology Division, Hospital de Clínicas de Porto Alegre, Rua Ramiro Barcelos, 2350, Building 12, Fourth floor, Porto Alegre, RS 90035-003 Brazil

**Keywords:** Metabolic syndrome, Insulin resistance, Insulin sensitivity

## Abstract

**Background:**

This study aimed to determine the ability of commonly used insulin resistance indices to identify the metabolic syndrome.

**Methods:**

183 people referred for outpatient care at the Metabolism Unit of Hospital de Clínicas de Porto Alegre were evaluated with anthropometric, blood pressure, lipid profile, and adiponectin measurements. Glucose tolerance status was determined by 2-h 75-g oral glucose tolerance test and glycosylated hemoglobin. Definition of metabolic syndrome was based on the Joint Interim Statement of different medical associations. Twenty-one indices of insulin resistance were estimated from published equations. The accuracy of these indices was determined by area under the ROC curve (AUC) analysis. In addition, we determined an optimal cut point for each index and its performance as a diagnostic test.

**Results:**

The study population was comprised of 183 people (73.2% women; 78.7% white; age 52.6 ± 12.0 years, mean ± standard deviation), of whom 140 (76.5%) had metabolic syndrome. The reciprocal of the Gutt index provided the greatest AUC for identification of metabolic syndrome, but there were no statistical differences between Gutt and 11 AUC indices. Gutt presented 86.4% sensitivity and 76.7% specificity to identify metabolic syndrome.

**Conclusions:**

A number of commonly employed indices of insulin resistance are capable of identifying individuals with the metabolic syndrome.

**Electronic supplementary material:**

The online version of this article (10.1186/s13098-018-0365-y) contains supplementary material, which is available to authorized users.

## Background

Insulin resistance is a condition in which a greater than normal amount of insulin is required to obtain a quantitatively normal metabolic response. Most commonly, insulin resistance is used to refer to the inability of insulin to stimulate glucose disposal [1]. Insulin resistance is also associated with the development of the metabolic syndrome [2], which represents a cluster of cardiometabolic risk factors which promote the development of cardiovascular disease and type 2 diabetes [3, 4]. The individual components of the metabolic syndrome include hyperglycemia, elevated blood pressure, elevated triglyceride levels, low HDL cholesterol levels, and central obesity [5]. A recent systematic review estimated the general prevalence of metabolic syndrome of adults in Brazil in 29.6% (range 14.9–65.3%) [6].

Although insulin resistance has been shown to be closely associated with the metabolic syndrome and to play an important physiopathological role in the development of the individual components of the metabolic syndrome [3], quantification of insulin resistance in general clinical practice is difficult. The most precise method for quantitating insulin resistance is the hyperinsulinemic euglycemic clamp technique, because it directly measures the effects of insulin to stimulate glucose utilization under steady-state conditions in vivo [7]. However, it involves intravenous infusion of insulin, frequent blood sampling over a 2-h period, and continuous adjustment of a glucose infusion, making it an impractical tool for large-scale epidemiological studies and clinical practice [8].

Consequently, a number of surrogate indices have been developed to estimate insulin sensitivity [8]. Both static indices, using fasting blood samples, and dynamic indices, requiring fasting and 2-h blood samples from an oral glucose tolerance test, have been developed [9, 10]. Measurements of adiponectin and inflammatory markers have also been used as a surrogate measurement of insulin sensitivity [8]. The performance of these equations has been evaluated in different populations against a variety of measures of insulin sensitivity (Additional file 1).

Insulin resistance is a key component of the metabolic syndrome, but it is not included as part of the definition because it is not easily quantitated in clinical practice. To investigate the relationship between insulin resistance and metabolic syndrome, we evaluated the correlation between multiple surrogate indices of insulin resistance and the presence of metabolic syndrome in adult participants, aged 24–83 years, with different degrees of glucose tolerance.

## Methods

### Subjects

The patient population initially consisted of 223 consecutive participants who did not have a previous diagnosis of metabolic syndrome and who were referred for outpatient care in the Metabolism Unit of Hospital de Clínicas de Porto Alegre. Forty participants were not included based on following exclusion criteria: insulin treatment, clinically significant autoimmune disease, uncompensated hypo- or hyperthyroidism, malignant disease that could affect 5-year survival, stage IV–V chronic kidney disease, AIDS, pregnancy/lactation, dementia, cirrhosis, hepatitis, glucocorticoid or anti-retroviral treatment, menopause hormone replacement therapy, and malnutrition. The remaining 183 participants were included in the analysis. Nine were on treatment with oral hypoglycemic agents. The research related to human use has been complied with all the relevant national regulations, institutional policies and in accordance to the tenets of the Helsinki Declaration, and has been approved by the authors’ institutional review board. The participants provided written informed consent prior to participation.

### Methods

Participants underwent a standard evaluation, which included medical history, physical examination, and anthropometric measurements. Ethnicity was based on self-reported skin color and recorded as white or non-white, which included black, brown, yellow, Indigenous, and undeclared, according to the national definition used in Brazil [11]. Physical activity was classified in four categories, based upon the classification proposed by Tuomilehto et al. [12]: sedentary, light exercise, moderate exercise, and heavy exercise. Waist circumference was taken at the midpoint between the lower costal margin and the iliac crest measured to the nearest 0.5 cm.

Blood pressure was measured in the seated position 1 week after withdrawal of all antihypertensive medications, in the right arm with an oscillometric monitor device (OMRON^®^ H-003D), with cuff adjusted for arm circumference. The mean of the last two measurements was used to estimate systolic and diastolic blood pressure.

Blood samples were drawn after a 12-h overnight fast for analysis of lipids (triglycerides, total, LDL and HDL cholesterol), high-sensitive C-reactive protein, and glycosylated hemoglobin (HbA1c). Triglyceride and HDL cholesterol measurements in participants who were receiving drug treatment for elevated plasma triglycerides levels and/or for reduced HDL cholesterol levels were not included in the analysis. At 8 am following a 10–12 h overnight fast, subjects received a 75-g oral glucose tolerance test with plasma glucose and insulin determinations at 0, 30, 60, 90 and 120 min. In the 9 subjects who were taking oral hypoglycemic agents, the oral glucose tolerance test was performed 4 days after withdrawal of hypoglycemic medications. Fasting glucose was defined by the glucose concentration at 0 min. All participants were classified according to glucose tolerance status [13] and presence of metabolic syndrome [5]. The methodology and procedures were reviewed to equate to STARD criteria, and the diagram to report flow of participants through the study is displayed in Additional file 2 [14].

### Classification of metabolic syndrome

Metabolic syndrome was defined as the presence of 3 of 5 following: waist circumference ≥ 80 cm for women and ≥ 94 cm for men; serum triglyceride ≥ 1.7 mmol/L) or receiving treatment for elevated serum triglycerides; HDL cholesterol < 1.0 mmol/L for men and < 1.3 mmol/L for women or receiving treatment for reduced HDL cholesterol; systolic blood pressure ≥ 130 mmHg or diastolic blood pressure ≥ 85 mmHg or receiving antihypertensive treatment; and fasting plasma glucose ≥ 6.1 mmol/L or receiving treatment for hyperglycemia, according to the Joint Interim Statement for the harmonization of metabolic syndrome criteria [5]. In addition, the American Diabetes Association criteria for impaired glucose tolerance (2 h-plasma glucose ≥ 7.8 mmol/L) was used as a criteria for hyperglycemia [13].

### Classification of glucose tolerance

Based on HbA1c and fasting and 2 h-plasma glucose concentrations, participants were categorized according to American Diabetes Association criteria as having normal glucose tolerance (fasting plasma glucose < 6.1 mmol/L, 2 h-plasma glucose level < 7.8 mmol/L, and HbA1c < 5.7% [39 mmol/mol]), impaired fasting glucose (fasting plasma glucose 6.1–6.9 mmol/L and 2 h-plasma glucose level < 7.8 mmol/L); impaired glucose tolerance (fasting plasma glucose < 6.1 mmol/L and 2 h-plasma glucose level 7.8–11.0 mmol/L), and diabetes (fasting plasma glucose ≥ 7.0 mmol/L and/or 2-h PG ≥ 11.1 mmol/L or HbA1c ≥ 6.5% [48 mmol/mol] or receiving medication for diabetes control) [13]. Participants with impaired fasting glucose and/or impaired glucose tolerance and/or HbA1c between 5.7% (39 mmol/mol) and 6.4% (48 mmol/mol) were considered to have prediabetes.

### Estimation of insulin resistance indices

Eleven static indices were analyzed: Bennet, fasting insulin (Ins_0min_), fasting insulin/fasting glucose ratio, fasting insulin resistance index (FIRI), fasting insulin sensitivity index (ISI_0min_), homeostasis model assessment (HOMA)-IR, HOMA-2-IR, HOMA-2-IS, McAuley, quantitative insulin sensitivity check index (QUICKI) and Raynaud. Eight dynamic indices were analyzed: Avignon, Gutt, Matsuda, oral glucose insulin sensitivity index (OGIS), Stumvoll with and without demographics, 2 h-insulin sensitivity index (ISI_120min_), and 2 h-insulin/2 h-glucose ratio.

All indices were calculated according to published equations, as described in Additional file 1. The reciprocal of an IS index was used as equivalent to an insulin resistance index (1/insulin sensitivity index = insulin resistance index). Two additional markers, adiponectin and HOMA-AD, were also examined because of their potential physiopathological association with metabolic syndrome [8]. Indices designed specifically to estimate beta-cell function were not considered.

### Statistical analysis

Data were expressed as absolute number and %, mean ± standard deviation or median (P25–P75). Sample size analysis was performed considering an expected sensitivity of 0.73 and an expected specificity of 0.70 to identify metabolic syndrome, based on a study that evaluated the performance of the HOMA index [15], and on an expected prevalence of metabolic syndrome of 0.30 [6]. It was estimated that 182 subjects would be necessary to achieve a precision of 0.118 for sensitivity and 0.080 for specificity. Calculations were made by using an Excel spreadsheet available online [16]. To compare demographic, clinical, and laboratory data, using the presence of metabolic syndrome in our sample, the Chi square test and independent-sample *t*-test were used as appropriate. Variables with a non-normal distribution were log transformed before analysis. The accuracy of insulin resistance indices to identify the metabolic syndrome was determined by analyzing the area under the curve (AUC) in a receiver operating characteristic (ROC) curve. The AUC comparison for different insulin resistance indices was examined by the method proposed by DeLong et al. [17]. For each index, we determined an optimal cut point at the ROC curve based on the Youden index and distance to coordinate (0, 1). The Youden index is a common summary measure of the ROC curve. It was calculated as (Sensitivity + Specificity) − 1 [18]. From this ideal cut point, we established the performance of these indices as a diagnostic test for the metabolic syndrome, including sensitivity, specificity, and positive and negative likelihood ratios. For practical clinical purposes, we estimated the probability of the outcome employing the Bayes nomogram. The pretest probability was defined as the prevalence of metabolic syndrome in our sample [19]. To deal with multiple testing, we performed a Bonferroni correction test [20]. The conventional *P* value of 0.05 was divided by the total number of analyzed indices in the present study (21 indices), giving a *P*-value of 0.0024 for statistical significance. For one-to-one comparisons, i.e., in Table 1, it was considered *P *< 0.05. Calculations were made by using SPSS (version 19.0; SPSS Inc., Chicago) and pROC package for R i386 (version 3.1.2.; R Foundation, Vienna).

**Table 1:** Participants’ demographic, clinical, and laboratory characteristics according to the presence of metabolic syndrome

	Metabolic syndrome		
	Absent	Present	*P* value^a^
Number (%)	43 (23.5)	140 (76.5)	–
Women *n* (%)	35 (81.4)	99 (70.7)	0.235
Age (years)	47.0 ± 12.8	54.1 ± 11.1	0.001^d^
White ethnicity *n* (%)^b^	34 (79.1)	110 (78.5)	0.811
Smoking *n* (%)	5 (11.6)	17 (12.1)	0.617
Physical activity^c^			0.057
Sedentary *n* (%)	16 (37.2)	78 (55.7)	–
Light exercise *n* (%)	18 (41.9)	42 (30.0)	–
Moderate exercise *n* (%)	6 (14.0)	18 (12.9)	–
Heavy exercise *n* (%)	3 (7.0)	2 (1.4)	–
BMI (kg/m^2^)	27.8 ± 5.1	32.3 ± 5.9	< 0.05^d^
Overweight *n* (%)	17 (39.5%)	50 (35.7%)	< 0.05^d^
Obesity *n* (%)	14 (32.6%)	83 (59.2%)	< 0.05^d^
Waist circumference (cm)			< 0.05^d^
Women	95.0 ± 13.4	103.0 ± 12.2	
Men	93.8 ± 11.4	107.0 ± 12.2	
Glucose tolerance status			< 0.001^d^
Normal glucose tolerance *n* (%)	39 (90.7)	19 (13.6)	
Prediabetes *n* (%)	3 (7.0)	78 (55.7)	
Type 2 diabetes *n* (%)	1 (2.3)	43 (30.7)	
Fasting plasma glucose (mmol/l)	5.0 (4.5–5.5)	5.8 (5.2–6.3)	< 0.05^d^
2-h plasma glucose (mmol/l)	6.1 (4.9–6.9)	9.5 (8.3–11.6)	< 0.05^d^
HbA1c (%)	5.5 (5.3–5.8)	6.2 (5.7–6.7)	< 0.001^d^
HbA1c (mmol/mol)	37 (34–40)	44 (39–50)	< 0.001^d^
HDL cholesterol (mmol/l)	1.35 (1.21–1.61)	1.19 (1.01–1.37)	< 0.05^d^
Triglycerides (mmol/l)	1.11 (0.78–1.52)	1.45 (1.11–1.99)	< 0.05^d^
High-sensitive C-reactive protein (nmol/l)	15.2 (5.7–31.4)	38.1 (12.4–80.0)	< 0.001^d^
Adiponectin (μg/mL)	16.5 (10.4–21.8)	11.0 (7.9–14.0)	0.001^d^
Systolic blood pressure (mm Hg)	123.5 (115–135)	142.5 (128.3–161.1)	< 0.05^d^
Diastolic blood pressure (mm Hg)	78.7 ± 11.2	87.6 ± 13.1	< 0.05^d^
Medications			
Antihypertensive *n* (%)	9 (20.9)	75 (53.5)	< 0.001^d^
Statin *n* (%)	4 (9.5)	24 (17.1)	0.325
Hypoglycemic *n* (%)	0 (0)	9 (6.4)	0.164
Insulin resistance indices			
2 h-insulin/2 h-glucose ratio	0.27 (0.40–0.70)	0.60 (0.37–0.96)	0.141
Fasting insulin (Ins_0min_)	7.7 (4.9–10.3)	12.5 (7.9–18.3)	< 0.001^b^
Fasting insulin/fasting glucose ratio	0.08 (0.05–0.10)	0.12 (0.07–0.17)	0.013^b^
FIRI	1.5 (1.0–2.2)	3.0 (1.8–4.3)	< 0.001^b^
HOMA-IR	1.6 (1.1–2.4)	3.3 (2.0–4.8)	< 0.001^b^
HOMA-2-IR	0.1 (0.1–0.2)	0.25 (0.20–0.40)	< 0.001^b^
Matsuda	2.2 (1.6–3.2)	3.6 (2.8–5.1)	< 0.001^b^
Insulin sensitivity indices			
Avignon	14.0 (9.2–26.2)	6.2 (4.1–9.6)	< 0.001^b^
Bennet	0.6 (0.5–0.7)	0.45 (0.39–0.55)	< 0.001^b^
Gutt	4.5 (3.7–5.4)	2.4 (2.1–3.2)	< 0.001^b^
HOMA-2-IS	692 (525–1081)	410 (278–654)	< 0.001^b^
ISI_0min_	15.1 (10.3–22.8)	7.5 (5.2–11.8)	< 0.001^b^
ISI_120min_	69.8 (27.7–40.4)	60.9 (37.2–95.9)	0.141
McAuley	8.3 (7.5–9.7)	7.1 (6.3–8.3)	0.001^b^
OGIS	422 (379–467)	325 (276–371)	< 0.001^b^
QUICKI	3.1 (3.0–3.4)	2.9 (2.8–3.1)	0.059
Raynaud	5.2 (3.9–8.2)	3.2 (2.2–5.0)	< 0.001^b^
Stumvoll with demographics	12.2 (9.6–15.2)	14.1 (2.2–27.8)	< 0.001^b^
Stumvoll without demographics	0.5 (0.5–1.0)	0.3 (0.25–0.50)	< 0.001^b^
Other indices			
Adiponectin	16.5 (10.0–22.9)	11.1 (8.2–14)	0.001^b^
HOMA-AD	2.4 (1.6–3.9)	6.1 (3.5–12.4)	< 0.001^b^

Data are expressed as the absolute number, % or mean ± standard deviation or median (P25–75)

^a^P value for comparisons between two groups was tested by χ^2^ test for categorical variables or Student’s *t*-test for continuous variables

^b^Ethnicity was recorded as white or non-white, which included black (*n *= 13), brown (*n *= 9), yellow (*n *= 0), indigenous (*n *= 3) and undeclared (*n *= 14)

^c^Participants reported the frequency of exercise in four categories, adapted from the classification proposed by Tuomilehto et al. [12]

^d^Significant statistical difference (*P *< 0.05)

## Results

### Participants’ characteristics

The patient population was subdivided by the absence (23.5%) or presence (76.5%) of metabolic syndrome. Participants with metabolic syndrome were older and had lower adiponectin plasma levels, and higher HbA1c and high-sensitive C-reactive protein levels. The groups did not differ by gender distribution, ethnicity, smoking habits, and physical activity status. As expected, the prevalence of prediabetes and type 2 diabetes was higher in the group with metabolic syndrome (Table 1).

### Accuracy of insulin resistance indices in the diagnosis of metabolic syndrome

**Fig. 1:**
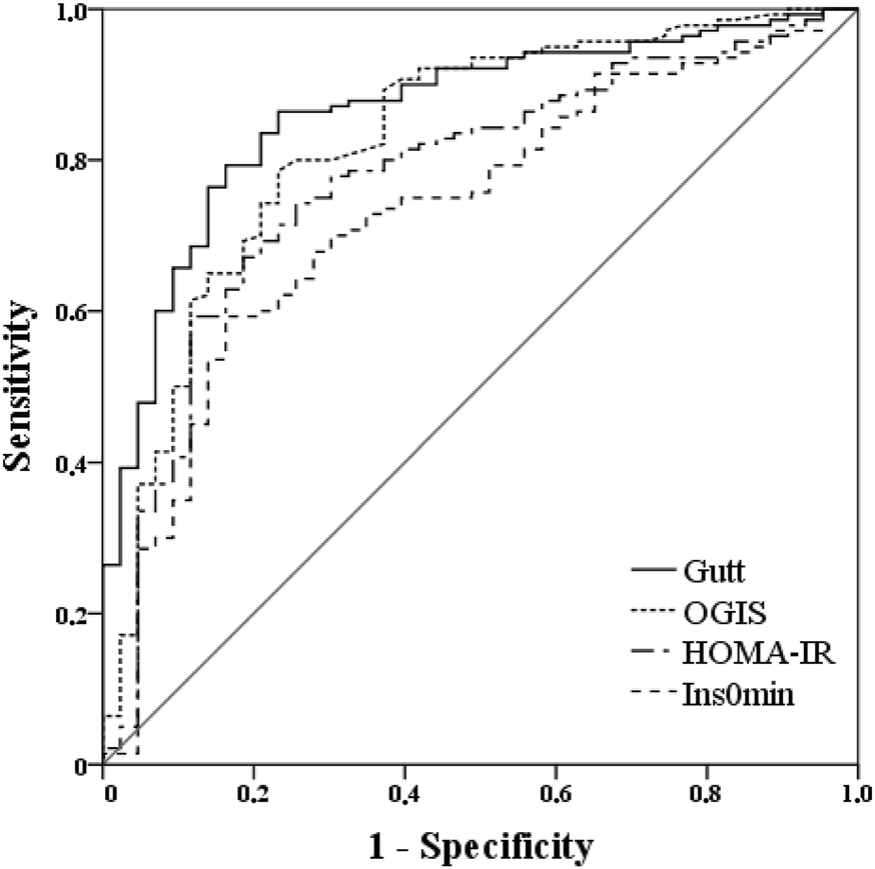
ROC curves of insulin resistance indices used to identify the metabolic syndrome. The two indices with greater area under the curve (AUC) in our analysis (Gutt and OGIS) and the two most frequently used indices in clinical practice and other research studies (HOMA-IR and fasting plasma insulin concentration) are displayed

**Fig. 2:**
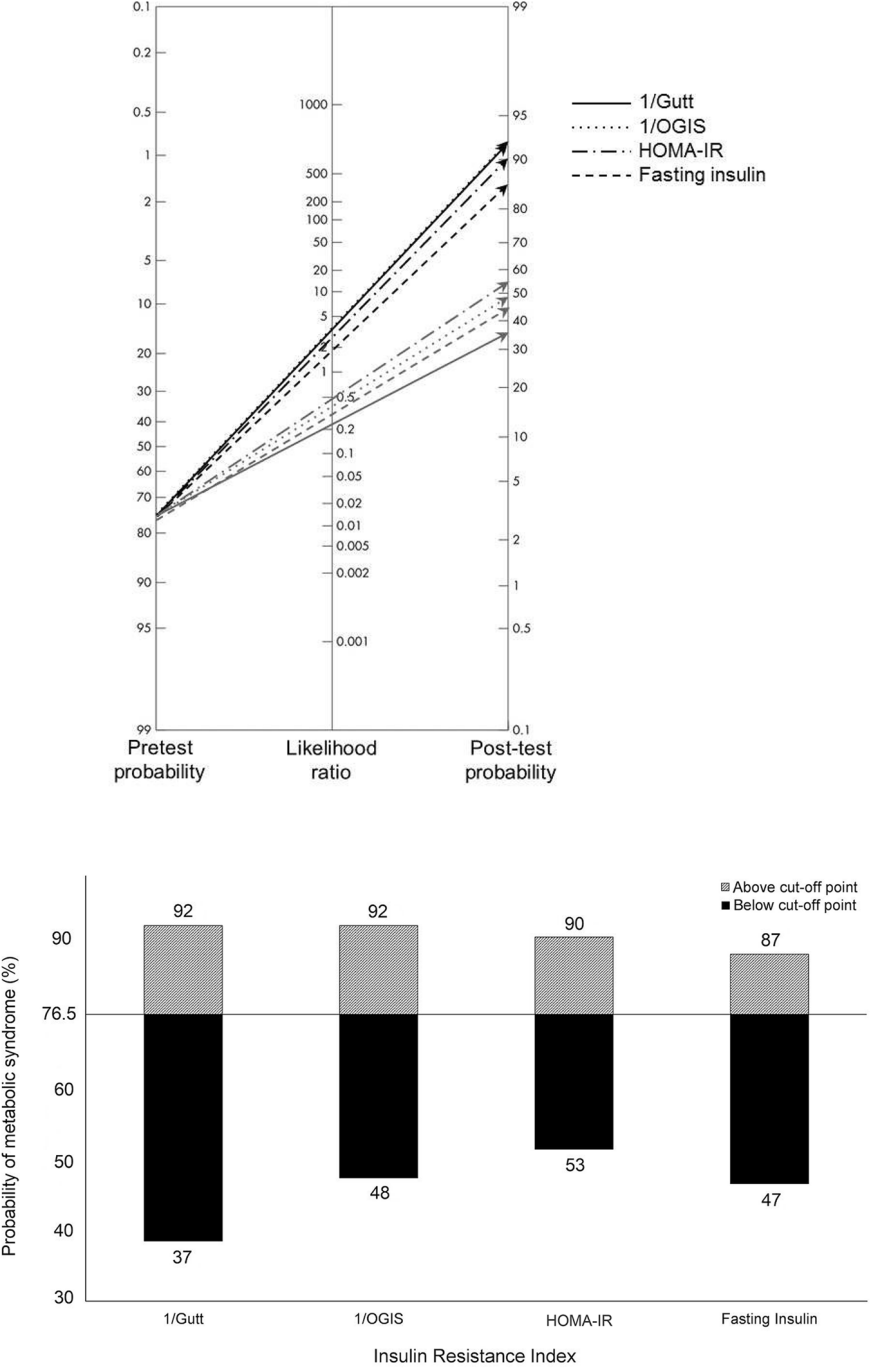
Fagan’s likelihood nomograms show the variation in probability of a metabolic syndrome diagnosis after a positive (above cut-off point) or negative test (below cut-off point) result for the reciprocals of Gutt and OGIS indices, as well as HOMA-IR and fasting insulin indices. The figure below synthesize these findings, comparing the variation of probability for metabolic syndrome between indices [Nomograms were adapted from Fagan TJ (N Engl J Med 1975;293:257; copyright 1975, New England Journal of Medicine, all rights reserved)]

ROC analysis showed that the reciprocal of Gutt and OGIS indices yielded an AUC above 0.8, whereas most other indices gave an AUC between 0.7 and 0.8 (Fig. 1). However, there was no statistical difference between the reciprocal of Gutt AUC and the following 11 indices of AUC (OGIS, Matsuda, HOMA-AD, Avignon, ISI_0min_, HOMA-IR, FIRI, Bennet, HOMA-2-IS, fasting insulin, and Raynaud; *P* ≥ 0.0024), indicating that these equations provide an index of insulin resistance that identifies individuals with the metabolic syndrome (Fig. 2; Additional file 3).

Since subjects with metabolic syndrome were older than subjects without the condition, we stratified our sample based on median of age and performed an additional analysis. For subjects younger than 53 years old, there was no statistical difference between the reciprocal of Gutt AUC and 16 indices of AUC; while for subjects older than 53 years old, there was no difference for 17 indices (Additional file 4).

Additionally, we performed a stratified analysis of AUC values for subjects with and without obesity and with normal and large waist circumference. Generally, Gutt still presented the largest AUC, but more indices were statistically equivalent to Gutt. When the subgroup of patients with obesity is analyzed, Gutt and OGIS present a great performance (AUC 0.930 and 0.924).

### Performance of insulin resistance indices as diagnostic tests for metabolic syndrome

Using the ideal cut point, we determined the sensitivity and specificity of each equation in demonstrating the presence of metabolic syndrome. The reciprocals of Gutt and OGIS, which had the greatest AUC values in predicting the metabolic syndrome, and HOMA-IR and fasting insulin, which are commonly employed indices for insulin resistance, are displayed in Fig. 2 and in Additional file 3. Using an optimal cut point of 0.268 for the reciprocal of Gutt, this index gave sensitivity of 86.4% (80.1–91.4%; CI 95%) and specificity of 76.7% (68.2–87.6%) to identify the metabolic syndrome. For a positive test result (test value above the cut point), the post test probability of identifying metabolic syndrome was 92, which means a 20% increase in the probability of identifying metabolic syndrome when Gutt has a positive result. For a negative test result (test value below the cut point), the post test probability was 37%, which means a 51% decrease in the probability of identifying metabolic syndrome when Gutt has a negative result. In comparison, for a positive HOMA-IR test result, the post test probability of identifying metabolic syndrome was 90% (increase of 17%); for a negative test result, the post test probability was 53% (decrease of 30%). The same procedure was applied to other indices (Fig. 2; Additional file 3).

## Discussion

In the present study, we evaluated the ability of all published indices of insulin resistance to identify the metabolic syndrome. From the quantitative standpoint, the Gutt index had the greatest area under the ROC curve (Fig. 1). However, 11 other indices, most notably the dynamic ones, were statistically non-inferior to the Gutt index. The inability to demonstrate statistically significant differences between those 12 indices of insulin resistance most likely is related to the conservative method utilized to define significance. Because 21 differences were evaluated, the alpha (P < 0.05) was split by 21, requiring a P < 0.0024 to demonstrate significance. If one-to-one comparisons are made using P < 0.05, the Gutt index outperforms all other indices, except OGIS. OGIS had a similar performance when the sample was stratified by age, BMI and waist circumference (Additional file 4). It should be noted that the Gutt index, although validated against the euglycemic insulin clamp, primarily included subjects who were obese and had normal glucose tolerance. Hanley et al. [21] found that the Gutt index demonstrated the best overall ability to predict type 2 diabetes compared to 18 other indices in a large multiethnic cohort. A Finnish study showed that Gutt was not statistically inferior to other indices when tested against the gold standard measure of insulin sensitivity obtained by the hyperinsulinemic euglycemic clamp [22].

Additionally, we found that adiponectin, an insulin sensibilizing hormone, and high-sensitive C-reactive protein, a marker of subclinical inflammation, were different in subjects with and without metabolic syndrome. The insulin sensitizer and anti-inflammatory role of adiponectin is a result of several actions: suppression of hepatic gluconeogenesis, activation of AMPK and fatty acid oxidation in skeletal muscle, suppression of the inflammatory response through the enhancement of nitric oxide synthase activity in endothelial cells and inhibition of the expression of adhesion molecules, such as VCAM-1, E-selectin, and ICAM [23]. In a previous study, we found that adiponectin levels decreased with increasing number of metabolic syndrome criteria, and it is in part determined by its relationship with abdominal adiposity [24]. Additionally, adiponectin levels were inversely related to HDL levels and high-sensitive C-reactive protein and positively related to blood pressure levels [25]. These findings were confirmed in an additional analysis within this study, where the number of metabolic syndrome criteria were greater in subjects more insulin resistant than those more insulin sensitive categorized by the median of insulin resistance indices (Additional file 5). From a physiopathological perspective, inflammation inhibits insulin action through the release of cytokines and adipocytokines and contributes to the progression from insulin resistance to the development of hyperglycemia [26], which explain the higher rates of dysglycemia and diabetes in the group with metabolic syndrome. These findings also corroborate the role of adipokines and inflammation in the pathogenesis of metabolic syndrome [25].

The present study has some limitations. First, our sample was composed by a predominantly White female population with a high prevalence of metabolic syndrome. It would be important to confirm these findings in further studies, in populations with a more diverse ethnic background, more balanced gender distribution, and a lower prevalence of metabolic syndrome. Second, methods for insulin measurement are not standardized [8]. Although we used a reliable assay with appropriate controls, these results might not be applicable to other studies, which use different assays to measure plasma insulin. Third, we did not perform euglycemic insulin clamps in the present study. It would have been of great interest to see how well the insulin clamp predicts the metabolic syndrome in comparison to the other 21 insulin resistance indices that were evaluated.

In summary, a variety of indices provide a measure of insulin resistance that can be used for clinical and epidemiological research studies. Because the equations used to estimate insulin resistance rely on different variables, i.e., hyperglycemia, hyperinsulinemia, and, in some cases, adiposity and demographic parameters, they reflect different components of the underlying physiopathological disturbances present in the metabolic syndrome. Thus, it is not surprising that there may be variability in their ability to predict the metabolic syndrome.

## Additional files

**Additional file 1.** Development and validation method of the equations for insulin resistance indices. This file contains a table that describes the development and validation method of each insulin resistance index. It also contains comments regarding their performance and applications in previous studies.

**Additional file 2.** Flow diagram. This diagram shows the flow of participants through the study.

**Additional file 3.** ROC curve analysis of insulin resistance indices used to identify metabolic syndrome/Performance of selected insulin resistance indices as diagnostic tests for metabolic syndrome (CI 95%). The first table shows the AUC values of insulin resistance indices to identify metabolic syndrome and their statistical comparison to the reciprocal of Gutt AUC, which had the best AUC. The second table shows the sensitivity, specificity, likelihood ratios and positive and negative predictive values of selected equations in identifying presence of metabolic syndrome.

**Additional file 4.** Performance of insulin resistance indices to identify metabolic syndrome in a stratified analysis by age, BMI and waist circumference. The tables show the performance of insulin resistance indices in subgroups stratified by age, BMI and waist circumference.

**Additional file 5.** Number of metabolic syndrome criteria by median of insulin sensitivity and resistance indices. The tables show a comparison between groups divided by median of selected insulin sensitivity and resistance indices for number of metabolic syndrome criteria.

## Abbreviations

AUC: area under the curve
FIRI: fasting insulin resistance index
HbA1c: glycosylated hemoglobin
HOMA: homeostasis model assessment
Ins_0min_: fasting insulin
ISI_0min_: fasting insulin sensitivity index
ISI_120min_: 2 h-insulin sensitivity index
OGIS: oral glucose insulin sensitivity index
QUICKI: quantitative insulin sensitivity check index
ROC: receiver operating characteristic
