# Supplementary material for: Accuracy of insulin resistance indices for metabolic syndrome: a cross-sectional study in adults
Source: Diabetol Metab Syndr. 2018 Aug 20;10:65. doi: 10.1186/s13098-018-0365-y (PMC6102896; doi:10.1186/s13098-018-0365-y)
Supplement: Supplementary file 1 — Additional file 1. Development and validation method of the equations for insulin resistance indices. This file contains a table that describes the development and validation method of each insulin resistance index. It also contains comments regarding their performance and applications in previous studies. [file 13098_2018_365_MOESM1_ESM.docx]

**Additional file 1 Development and validation method of the equations for insulin resistance indices**

| **Insulin resistance indices** | **Formula** | **Development/Validation method/Population** | **Comments** |
| --- | --- | --- | --- |
| **Static indices** |  |  |  |
| 1/**Bennet [1]** | 1/(**1/(log Ins_0min_× log Glu_0min_**) | Derived from frequently sampled intravenous glucose tolerance test (FSIVGTT). | Performed similarly to fasting insulin and to the mean of two fasting insulin measurements. |
| **Fasting insulin (Ins_0min_) [2]** | **Ins_0min_** | Correlated with hyperinsulinemic euglycemic clamp. 132 subjects with different degrees of glucose tolerance. | Common marker to evaluate insulin resistance in clinical and epidemiological studies. |
| **Fasting insulin/fasting  glucose ratio [3]** | **Ins_0min_/Glu_0min_** | Derived from Ins_0min_ and Glu_0min_ values. Correlated with hyperinsulinemic euglycemic clamp. 469 Pima Indians without type 2 diabetes. | May conceal subjects in whom insulin and glucose rise simultaneously, albeit the latter may be within the "normal" range [6]. |
| **Fasting insulin resistance index (FIRI) [4]** | **(Ins_0min_ × Glu_0min_)/25** | Product of Ins_0min_ and Glu_0min_, which was normalized to an expected glucose value of 5 mmol/L and an insulin value of 5 mU/L to give a reference range centered on unity. Correlated strongly with minimal model of Bergman et al. for 13 normal subjects. | Correlated positively with triglyceride and negatively with HDL cholesterol in 30 subjects newly diagnosed with type 2 diabetes. Near-perfect linear relation with HOMA-IR [4]. |
| **Homeostasis model assessment (HOMA-IR) [5]** | **(Ins_0min_ × Glu_0min_)/22.5** | Quantitative assessment of the contributions of insulin resistance and deficient beta-cell function to the fasting hyperglycemia based on predictions from computed-solved model and its comparison with patients’ fasting values. Correlated with hyperinsulinemic euglycemic clamp and FSIVGTT. Six normal subjects and nine normal-weight patients with type 2 diabetes. | Most-used index to classify insulin resistance in clinical and epidemiological studies. Estimates from the model have low precision (31% as coefficient of variation for insulin resistance). |
| **HOMA-2-IR [6]** | Calculated with a spreadsheet [http://www.dtu.ox.ac.uk; accessed October 17, 2015]. | Updated HOMA model that takes account of variations in hepatic and peripheral glucose resistance, increases in the insulin secretion curve for plasma glucose concentrations above 10 mmol/L, and the contribution of circulating proinsulin. | The model was recalibrated also to give % values of 100% in normal young adults when using currently available assays for insulin, specific insulin, or C-peptide. |
| 1/**HOMA-2-IS [5]** |  |  |  |
| 1/**Fasting insulin sensitivity index (ISI_0min_) [7]** | **10,000/(Ins_0min_× Glu_0min_)** | Based on a dose-response curve of insulin to glucose. Compared with two consecutive 100g oral glucose tolerance test (OGTT) performed on 14 men of normal weight without a family history of diabetes. |  |
| 1/**McAuley [8]** | **1/(e^[2.63— 0.28ln(Ins0min— 0.31ln(Tg0min)]^)** | Comparison of several laboratorial measurements and indices to predict insulin sensitivity; fasting insulin and fasting triglycerides were the most accurate variables. Compared with hyperinsulinemic euglycemic clamp; 178 normoglycemic individuals. | Index application, rather than the use of fasting insulin alone, resulted in a higher sensitivity and a maintained specificity when predicting insulin sensitivity. |
| 1/**Quantitative insulin sensitivity  check index (QUICKI) [9]** | 1/(**1/(log Ins_0min_+ log Glu_0min_)**) | Derived from hyperinsulinemic euglycemic glucose clamp and insulin modified FSIVGTT; 56 subjects. |  |
| 1/**Raynaud [10]** | 1/(**40/Ins_0min_**) | Adjustment for Ins_0min_ value according to the hyperbolic relationship between Ins_0min_ and IS, proposed by Kahn et al. [11]. Compared with intravenous glucose tolerance test for calculation of SI by the minimal model (FSIGVTT). 70 subjects without type 2 diabetes, mainly overweight. | Better prediction of minimal model-derived IS than HOMA-IR, Ins_0min_^,^ and Ins_0min_/Glu_0min_ ratio. |
| **Dynamic indices** |  |  |  |
| **2h-insulin/2h-glucose ratio [3]** | **Ins_120min_/Glu_120min_** | Derived from OGTT. Correlated with hyperinsulinemic euglycemic clamp. 469 Pima Indians without type 2 diabetes. |  |
| 1/**Avignon [12]** | 1/{[**(w × Sib) + Si_2h_] / 2**}† | Derived from OGTT. Compared with FSIVGTT and HOMA-IR; 47 subjects with different degrees of glucose tolerance. |  |
| 1/**Gutt [13]** | 1/((**m/MPG) / log MSI)*** | Adapted from the sensitivity index developed by Cederholm et al. [14]. Correlated with the index obtained from hyperinsulinemic euglycemic clamp when applied prospectively in comparative studies; 135 subjects, mainly obese and normoglycemic. | Best overall ability to predict diabetes in a large, multiethnic cohort [1]. |
| 1/**2h-insulin sensitivity index (ISI_120min_) [7]** | **10,000/(Ins_120min_× Glu_120min_)** | Similar to ISI**_0min._** |  |
| 1/**Matsuda [15]** | **10,000/√ (Glu_0min_ × Ins_0min_) (mG_a_ × m I_a_)** | Derived from OGTT. Correlated with hyperinsulinemic euglycemic clamp; 153 subjects with different degrees of glucose tolerance. | Represents both hepatic and peripheral tissue sensitivity to insulin [http://www.dtu.ox.ac.uk; accessed October 17, 2015]. |
| 1/**Oral glucose insulin sensitivity index (OGIS) [16]** | Calculated with a spreadsheet. [http://webmet.pd.cnr.it/ogis/download.php; accessed October 17, 2015] | Derived from OGTT. Compared with hyperinsulinemic euglycemic clamp; 91 subjects with different degrees of glucose tolerance. Additional testing was performed on an independent group of 13 subjects with impaired glucose tolerance. |  |
| 1/**Stumvoll with demographics [17]** | **0.222 – 0.00333 × BMI – 0.0000779 × Ins_120min_ – 0.000422 × age** | Derived from the OGTT. Correlated with hyperinsulinemic euglycemic clamp; 104 white subjects without type 2 diabetes. | Uses demographic data such as age, sex, and BMI, along with plasma glucose and insulin to IS [20]. |
| 1/**Stumvoll without demographics [17]** | **0.156 – 0.0000459 × Ins_120min_– 0.0000321 × Ins_0min_ – 0.00541 × Glu_0min_** |  |  |
| **Additional indices** |  |  |  |
| 1/**Adiponectin [18]** | Serum adiponectin | Correlated directly with whole-body insulin sensitivityin patients with obesity and type 2 diabetes. Compared with hyperinsulinemic euglycemic clamp; 117 Japanese subjects with various degrees of glucose tolerance. | M-value correlates significantly and independently with adiponectin levels. |
| **HOMA-AD [19]** | **(Ins_0min_× Glu_0min_)/ adiponectin** | Proposed correction to HOMA-IR using adiponectin levels. Compared with hyperinsulinemic euglycemic clamp; 117 Japanese subjects with various degrees of glucose tolerance. | M-values from hyperinsulinemic euglycemic clamp were significantly more correlated with HOMA-AD than with HOMA-IR values. |

*m = (75,000 mg + [fasting glucose — 2-h glucose] × 0.19 × weight) / 120 min; MPG = mean of fasting and 2-h glucose concentrations (mg/dl); MSI = mean of fasting and 2-h insulin concentrations (mU/l). †w = mean Si2h / mean Sib, Sib = 108 / (fasting insulin [µU/ml] × fasting glucose [mg/dl] × VD), Si2h = 108 / (2-h insulin [µU/ml] × 2-h glucose [mg/dl] × VD), where VD = 150 ml/kg × weight. BMI, body mass index;Glu_0min_, fasting glucose; Glu_0min_, 2h- glucose; G_a_, glucose area; Ins_0min_, fasting insulin; Ins_120min_, 2-h insulin; I_a_, insulin area; ln, natural logarithm; mG_a_, mean glucose area; m Ia, mean insulin area; Tg, serum triglycerides. The formulas were computed with the units for glucose, insulin, and triglycerides described in the original articles.

**References**

[1] Anderson R, Hamman R, Savage P, et al. Exploration of simple insulin sensitivity measures derived from frequently sampled intravenous glucose tolerance (FSIGT) tests. The Insulin Resistance Atherosclerosis Study. Am J Epidemiol. 1995;142 724-32.

[2] Laakso M. How Good a Marker Is Insulin Level for Insulin Resistance? Am J Epidemiol 1993;137(9):959–65.

[3] Hanson RL, Pratley RE, Bogardus C, Narayan KMV, Roumain JML, Imperatore G, et al. Evaluation of Simple Indices of Insulin Sensitivity and Insulin Secretion for Use in Epidemioiogic Studies. Am J Epidemiol 2000; 151(2):190–8.

[4] Duncan M, Singh B, Wise P, Carter G, Alaghband-Zadeh J. A simple measure of insulin resistance. The Lancet. 1995; 346(8967):120–1.

[5] Matthews DR, Hosker JP, Rudenski AS, Naylor BA, Treacher DF, Turner RC. Homeostasis model assessment: insulin resistance and ?-cell function from fasting plasma glucose and insulin concentrations in man. Diabetologia. 1985; 28(7):412–9.

[6] Levy JC, Matthews DR, Hermans MP. Correct Homeostasis Model Assessment (HOMA) Evaluation Uses the Computer Program. Diabetes Care. 1998; 21(12):2191–2.

[7] Sluiter WJ, Erkelens DW, Terpstra P, Reitsma WD, Doorenbos H. Glucose Tolerance and Insulin Release, A Mathematical Approach: II. Approximation of the Peripheral Insulin Resistance After Oral Glucose Loading. Diabetes. 1976; 25(4):245–9.

[8] Mcauley KA, Williams SM, Mann JI, Walker RJ, Lewis-Barned NJ, Temple LA, et al. Diagnosing Insulin Resistance in the General Population. Diabetes Care. 2001;24(3):460–4.

[9] Katz A, Nambi SS, Mather K, Baron AD, Follmann DA, Sullivan G, et al. Quantitative Insulin Sensitivity Check Index: A Simple, Accurate Method for Assessing Insulin Sensitivity In Humans. J Clin Endocrinol Metab. 2000; doi: 10.1210/jcem.85.7.6661.

[10] Raynaud E, Perez-Martin A, Brun JF, Benhaddad AA, Mercier J. Revised concept for the estimation of insulin sensitivity from a single sample. Diabetes Care. 1999;22(6):1003–4.

[11] Kahn SE, Prigeon RL, Mcculloch DK, Boyko EJ, Bergman RN, Schwartz MW, et al. Quantification of the relationship between insulin sensitivity and beta-cell function in human subjects. Evidence for a hyperbolic function. Diabetes. 1993;42(11):1663–72.

[12] Avignon A, Bœgner C, Mariano-Goulart D, Colette C, Monnier L. Assessment of insulin sensitivity from plasma insulin and glucose in the fasting or post oral glucose-load state. Int J Obes. 1999;23(5):512–7.

[13] Gutt M, Davis CL, Spitzer SB, Llabre MM, Kumar M, Czarnecki EM, et al.: Validation of the insulin sensitivity index (ISI0,120): comparison with other measures. Diabetes Res Clin Pr. 2000; 47(3):177–84.

[14] Cederholm J, Wibell L. Insulin release and peripheral sensitivity at the oral glucose tolerance test. Diabetes Res Clin Pr 1990;10(2):167–75.

[15] Matsuda M, Defronzo RA. Insulin sensitivity indices obtained from oral glucose tolerance testing: comparison with the euglycemic insulin clamp. Diabetes Care. 1999;22(9):1462–70.

[16] Mari A, Pacini G, Murphy E, Ludvik B, Nolan JJ. A Model-Based Method for Assessing Insulin Sensitivity From the Oral Glucose Tolerance Test. Diabetes Care. 2001;24(3):539–48.

[17] Stumvoll M, Mitrakou A, Pimenta W, Jenssen T, Yki-Jarvinen H, Haeften TV, et al. Use of the oral glucose tolerance test to assess insulin release and insulin sensitivity. Diabetes Care. 2000;23(3):295-301.

[18] Weyer C. Hypoadiponectinemia in Obesity and Type 2 Diabetes: Close Association with Insulin Resistance and Hyperinsulinemia. J Clin Endocrinol Metab. 2001; doi: 10.1210/jcem.86.5.7463.

[19] Matsuhisa M, Yamasaki Y, Emoto M, Shimabukuro M, Funahashi T, Matsuzawa Y. A novel index of insulin resistance determined from the homeostasis model assessment index and adiponectin levels in Japanese subjects. Diabetes Res Clin Pr. 2007; doi: 10.1016/j.diabres.2006.10.005.

[20] Liu R, Christoffel KK, Brickman WJ, Liu X, Gadgil M, Wang G, et al. Do static and dynamic insulin resistance indices perform similarly in predicting pre-diabetes and type 2 diabetes? Diabetes Res Clin Pr. 2014; doi: 10.1016/j.diabres.2014.04.014.
