# Supplementary figures and images for: Accuracy of insulin resistance indices for metabolic syndrome: a cross-sectional study in adults
Source: Diabetol Metab Syndr. 2018 Aug 20;10:65. doi: 10.1186/s13098-018-0365-y (PMC6102896; doi:10.1186/s13098-018-0365-y)

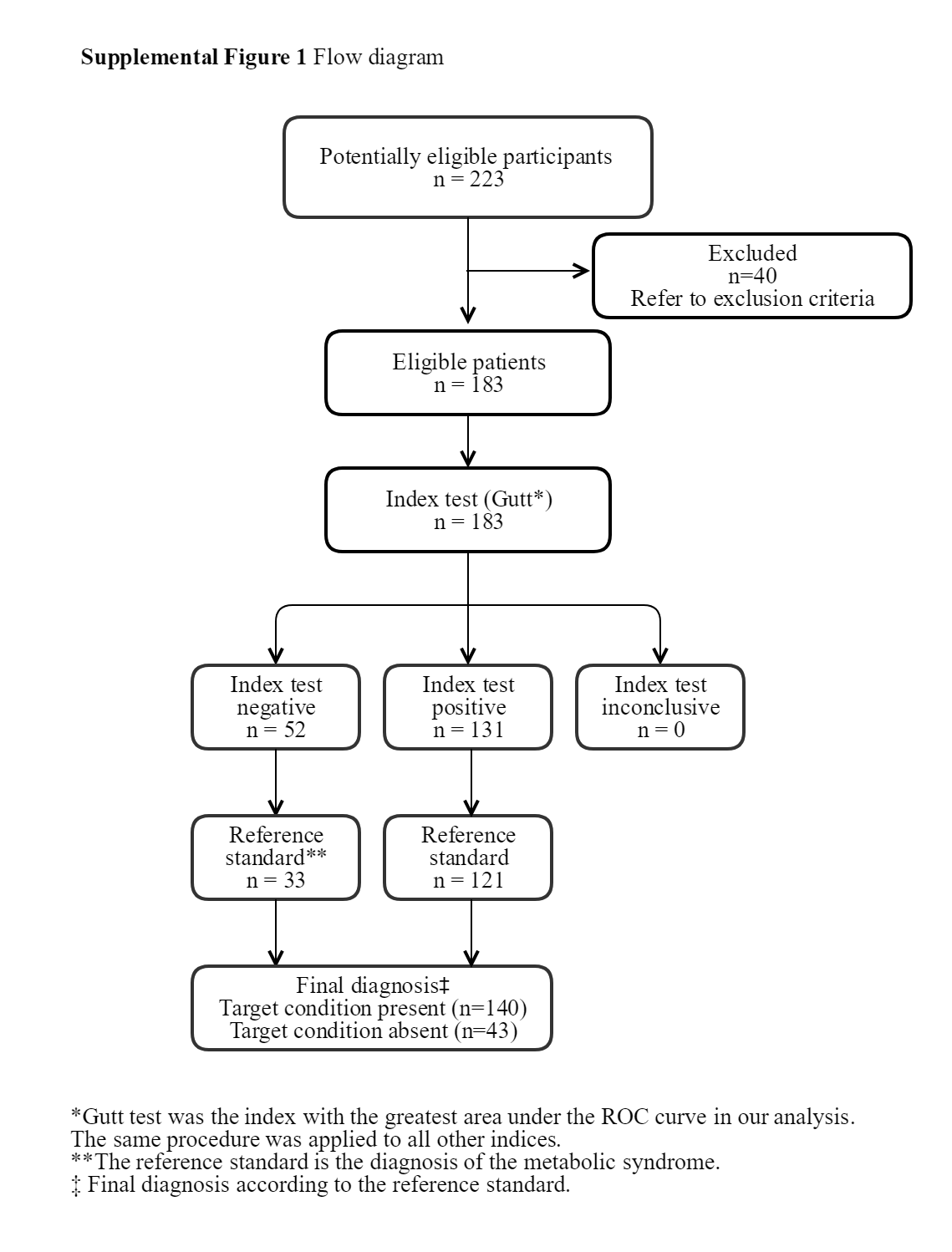

Supplement: Supplementary file 2 — Additional file 2. Flow diagram. This diagram shows the flow of participants through the study. [file 13098_2018_365_MOESM2_ESM.tiff]
