# Supplementary material for: Accuracy of insulin resistance indices for metabolic syndrome: a cross-sectional study in adults
Source: Diabetol Metab Syndr. 2018 Aug 20;10:65. doi: 10.1186/s13098-018-0365-y (PMC6102896; doi:10.1186/s13098-018-0365-y)
Supplement: Supplementary file 3 — Additional file 3. ROC curve analysis of insulin resistance indices used to identify metabolic syndrome/Performance of selected insulin resistance indices as diagnostic tests for metabolic syndrome (CI 95%). The first table shows the AUC values of insulin resistance indices to identify metabolic syndrome and their statistical comparison to the reciprocal of Gutt AUC, which had the best AUC. The second table shows the sensitivity, specificity, likelihood ratios and positive and negative predictive values of selected equations in identifying presence of metabolic syndrome. [file 13098_2018_365_MOESM3_ESM.docx]

**Additional file 3**

1. **ROC curve analysis of insulin resistance indices used to identify metabolic syndrome**

| Insulin resistance index | AUC | 95% CI | | *P*^a^ | Sensitivity | Specificity |
| --- | --- | --- | --- | --- | --- | --- |
| 1/Gutt^D^ | 0.864 | 0.805 | 0.924 | - | 86.4 | 76.7 |
| 1/Oral glucose insulin sensitivity index (OGIS)^D^ | 0.828 | 0.754 | 0.902 | 0.235 | 78.6 | 76.7 |
| 1/Matsuda^D^ | 0.790 | 0.709 | 0.870 | 0.042 | 75.0 | 76.7 |
| HOMA-AD | 0.789 | 0.703 | 0.874 | 0.137 | 72.0 | 80.0 |
| 1/Avignon^D^ | 0.779 | 0.699 | 0.859 | 0.053 | 69.3 | 81.4 |
| 1/Fasting insulin sensitivity index (ISI_0min_)^S^ | 0.778 | 0.698 | 0.858 | 0.039 | 75.0% | 74.4% |
| Homeostatic model assessment (HOMA)-IR^S^ | 0.776 | 0.696 | 0.856 | 0.035 | 73.4 | 74.4 |
| Fasting insulin resistance index (FIRI)^S^ | 0.776 | 0.696 | 0.856 | 0.035 | 74.3 | 74.4 |
| 1/Bennet^S^ | 0.757 | 0.675 | 0.839 | 0.014 | 71.4 | 76.7 |
| 1/HOMA-2-IS^S^ | 0.755 | 0.674 | 0.837 | 0.014 | 71.4 | 74.4 |
| Fasting insulin^S^ | 0.731 | 0.646 | 0.816 | 0.003 | 83.7 | 53.9 |
| 1/Raynaud^S^ | 0.731 | 0.646 | 0.816 | 0.003 | 59.3 | 83.7 |
| 1/McAuley^S^ | 0.724 | 0.639 | 0.809 | 0.002 ^c^ | 59.3 | 83.7 |
| HOMA-2-IR^S^ | 0.719 | 0.636 | 0.802 | 0.001 ^c^ | 50.0 | 86.0 |
| 1/Adiponectin | 0.685 | 0.576 | 0.793 | 0.004 ^c^ | 84.8 | 54.3 |
| 1/Stumvoll without demographics^D^ | 0.683 | 0.606 | 0.760 | <0.001 ^c^ | 59.3 | 83.7 |
| Quantitative insulin sensitivity check index (QUICKI)^S^ | 0.663 | 0.571 | 0.756 | <0.001^†^ | 58.6 | 74.4 |
| Fasting insulin/fasting glucose ratio^S^ | 0.663 | 0.573 | 0.753 | <0.001 ^c^ | 58.6 | 74.4 |
| 2h-insulin/2h-glucose ratio^D^ | 0.625 | 0.534 | 0.717 | <0.001 ^c^ | 35.7 | 88.4 |
| 1/2h-insulin sensitivity index (ISI_120min_)^D^ | 0.625 | 0.534 | 0.717 | <0.001 ^c^ | 35.7 | 88.4 |
| 1/Stumvoll with demographics^D^ | 0.558 | 0.477 | 0.638 | <0.001 ^c^ | 42.1 | 88.4 |

All AUCs were compared with the reciprocal of Gutt index AUC.
^S^, static index; ^D^, dynamic index; CI, confidence interval
^a^ *p* value indicates AUC comparison *versus* 1/Gutt AUC.

^b^ This cut point was defined for the formula as an insulin resistance index because it was used in our statistical analysis.

^c^Significant statistical difference (*p*<0.0024).

**2. Performance of selected insulin resistance indices as diagnostic tests for metabolic syndrome (CI 95%)**

| **IRI** | **Youden index** | **Cut point** | **Sensitivity (%)** | **Specificity (%)** | **Positive LR*** | **Negative LR** | **Positive predictive value (%)** | **Negative predictive value (%)** |
| --- | --- | --- | --- | --- | --- | --- | --- | --- |
| **1/Gutt** | 0.631 | 0.268 | 86.4 (80.1–91.4) | 76.7 (62.8–87.6) | 3.71 (2.15–6.40) | 0.18 (0.11–0.28) | 92 (87–95) | 37 (26–48) |
| **1/OGIS** | 0.553 | 0.0026 | 78.6 (71.3–84.8) | 76.7 (62.8–87.6) | 3.37 (1.95–5.84) | 0.28 (0.20–0.40) | 92 (86–95) | 48 (39–57) |
| **HOMA-IR** | 0.487 | 2.122 | 73.4 (66.6–81.0) | 74.4 (60.2–85.8) | 2.90 (1.73–4.88) | 0.35 (0.25–0.48) | 90 (85–94) | 53 (45–61) |
| **Ins_0min_** | 0.430 | 10.735 | 83.7 (70.9–92.7) | 53.9 (51.0–67.2) | 2.06 (1.42–2.97) | 0.27 (0.18–0.43) | 87 (82–91) | 47 (37–58) |

*LR = likelihood ratio.
