# Supplementary material for: Accuracy of insulin resistance indices for metabolic syndrome: a cross-sectional study in adults
Source: Diabetol Metab Syndr. 2018 Aug 20;10:65. doi: 10.1186/s13098-018-0365-y (PMC6102896; doi:10.1186/s13098-018-0365-y)
Supplement: Supplementary file 4 — Additional file 4. Performance of insulin resistance indices to identify metabolic syndrome in a stratified analysis by age, BMI and waist circumference. The tables show the performance of insulin resistance indices in subgroups stratified by age, BMI and waist circumference. [file 13098_2018_365_MOESM4_ESM.docx]

**Additional file 4 Performance of insulin resistance indices to identify metabolic syndrome in a stratified analysis by age, BMI and waist circumference**

**Age**

1. **Performance in younger subjects (age < 53 years) (n=89):**

| Insulin resistance index | AUC | 95% CI | | *P* value^a^ |
| --- | --- | --- | --- | --- |
| 1/Gutt^D^ | 0.866 | 0.769 | 0.933 | - |
| 1/Oral glucose insulin sensitivity index (OGIS)^D^ | 0.857 | 0.758 | 0.927 | 0.821 |
| 1/Matsuda^D^ | 0.806 | 0.699 | 0.888 | 0.248 |
| HOMA-AD | 0.813 | 0.707 | 0.893 | 0.363 |
| 1/Avignon^D^ | 0.805 | 0.699 | 0.887 | 0.359 |
| 1/Fasting insulin sensitivity index (ISI_0min_)^S^ | 0.805 | 0.698 | 0.887 | 0.129 |
| Homeostatic model assessment (HOMA)-IR^S^ | 0.770 | 0.669 | 0.853 | 0.322 |
| Fasting insulin resistance index (FIRI)^S^ | 0.770 | 0.669 | 0.853 | 0.322 |
| 1/Bennet^S^ | 0.748 | 0.645 | 0.834 | 0.055 |
| 1/HOMA-2-IS^S^ | 0.754 | 0.651 | 0.839 | 0.077 |
| Fasting insulin^S^ | 0.712 | 0.606 | 0.803 | 0.016 |
| 1/Raynaud^S^ | 0.742 | 0.629 | 0.836 | 0.016 |
| 1/McAuley^S^ | 0.732 | 0.618 | 0.827 | 0.053 |
| HOMA-2-IR^S^ | 0.724 | 0.610 | 0.821 | 0.032 |
| 1/Adiponectin | 0.698 | 0.582 | 0.798 | 0.016 |
| 1/Stumvoll without demographics^D^ | 0.718 | 0.604 | 0.816 | 0.014 |
| Quantitative insulin sensitivity check index (QUICKI)^S^ | 0.862 | 0.773 | 0.926 | 0.011 |
| Fasting insulin/fasting glucose ratio^S^ | 0.646 | 0.583 | 0.745 | 0.001^b^ |
| 2h-insulin/2h-glucose ratio^D^ | 0.650 | 0.541 | 0.748 | <0.001^b^ |
| 1/2h-insulin sensitivity index (ISI_120min_)^D^ | 0.593 | 0.484 | 0.696 | <0.001^b^ |
| 1/Stumvoll with demographics^D^ | 0.504 | 0.396 | 0.612 | <0.001^b^ |

1. **Performance in older subjects (age ≥ 53 years) (n=94):**

| Insulin resistance index | AUC | 95% CI | | *P* value^a^ |
| --- | --- | --- | --- | --- |
| 1/Gutt^D^ | 0.866 | 0.780 | 0.927 | - |
| 1/Oral glucose insulin sensitivity index (OGIS)^D^ | 0.787 | 0.684 | 0.869 | 0.405 |
| 1/Matsuda^D^ | 0.859 | 0.708 | 0.886 | 0.896 |
| HOMA-AD | 0.837 | 0.740 | 0.909 | 0.948 |
| 1/Avignon^D^ | 0.804 | 0.703 | 0.883 | 0.897 |
| 1/Fasting insulin sensitivity index (ISI_0min_)^S^ | 0.779 | 0.676 | 0.862 | 0.570 |
| Homeostatic model assessment (HOMA)-IR^S^ | 0.836 | 0.746 | 0.905 | 0.570 |
| Fasting insulin resistance index (FIRI)^S^ | 0.836 | 0.746 | 0.905 | 0.570 |
| 1/Bennet^S^ | 0.818 | 0.725 | 0.890 | 0.427 |
| 1/HOMA-2-IS^S^ | 0.810 | 0.716 | 0.884 | 0.381 |
| Fasting insulin^S^ | 0.798 | 0.702 | 0.874 | 0.306 |
| 1/Raynaud^S^ | 0.730 | 0.622 | 0.821 | 0.306 |
| 1/McAuley^S^ | 0.724 | 0.616 | 0.816 | 0.268 |
| HOMA-2-IR^S^ | 0.693 | 0.583 | 0.789 | 0.156 |
| 1/Adiponectin | 0.767 | 0.663 | 0.853 | 0.493 |
| 1/Stumvoll without demographics^D^ | 0.644 | 0.532 | 0.746 | <0.001^b^ |
| Quantitative insulin sensitivity check index (QUICKI)^S^ | 0.734 | 0.633 | 0.820 | 0.129 |
| Fasting insulin/fasting glucose ratio^S^ | 0.740 | 0.639 | 0.825 | <0.001^b^ |
| 2h-insulin/2h-glucose ratio^D^ | 0.686 | 0.582 | 0.778 | 0.019 |
| 1/2h-insulin sensitivity index (ISI_120min_)^D^ | 0.686 | 0.582 | 0.778 | 0.019 |
| 1/Stumvoll with demographics^D^ | 0.636 | 0.531 | 0.733 | <0.001^b^ |

**BMI**

1. **Performance in subjects without obesity (n=87)**

| Insulin resistance index | AUC | 95% CI | | *P* value^a^ |
| --- | --- | --- | --- | --- |
| 1/Gutt^D^ | 0.803 | 0.695 | 0.912 | 0.658 |
| 1/Oral glucose insulin sensitivity index (OGIS)^D^ | 0.787 | 0.676 | 0.898 | 0.127 |
| 1/Matsuda^D^ | 0.729 | 0.606 | 0.851 | 0.344 |
| HOMA-AD | 0.744 | 0.625 | 0.863 | 0.277 |
| 1/Avignon^D^ | 0.740 | 0.623 | 0.858 | 0.232 |
| 1/Fasting insulin sensitivity index (ISI_0min_)^S^ | 0.738 | 0.622 | 0.854 | 0.230 |
| Homeostatic model assessment (HOMA)-IR^S^ | 0.738 | 0.622 | 0.854 | 0.481 |
| Fasting insulin resistance index (FIRI)^S^ | 0.738 | 0.622 | 0.854 | 0.481 |
| 1/Bennet^S^ | 0.690 | 0.563 | 0.816 | 0.174 |
| 1/HOMA-2-IS^S^ | 0.678 | 0.547 | 0.808 | 0.188 |
| Fasting insulin^S^ | 0.650 | 0.516 | 0.783 | 0.068 |
| 1/Raynaud^S^ | 0.650 | 0.516 | 0.783 | 0.021 |
| 1/McAuley^S^ | 0.631 | 0.497 | 0.765 | 0.013 |
| HOMA-2-IR^S^ | 0.616 | 0.480 | 0.751 | 0.008 |
| 1/Adiponectin | 0.651 | 0.517 | 0.785 | 0.061 |
| 1/Stumvoll without demographics^D^ | 0.722 | 0.603 | 0.841 | 0.077 |
| Quantitative insulin sensitivity check index (QUICKI)^S^ | 0.583 | 0.441 | 0.724 | 0.012 |
| Fasting insulin/fasting glucose ratio^S^ | 0.569 | 0.430 | 0.709 | 0.007 |
| 2h-insulin/2h-glucose ratio^D^ | 0.530 | 0.396 | 0.665 | p<0.001^b^ |
| 1/2h-insulin sensitivity index (ISI_120min_)^D^ | 0.530 | 0.396 | 0.665 | p<0.001^b^ |
| 1/Stumvoll with demographics^D^ | 0.632 | 0.508 | 0.755 | 0.007 |

1. **Performance in subjects with obesity (n=96):**

| Insulin resistance index | AUC | 95% CI | | *P* value^a^ |
| --- | --- | --- | --- | --- |
| 1/Gutt^D^ | 0.930 | 0.875 | 0.985 | - |
| 1/Oral glucose insulin sensitivity index (OGIS)^D^ | 0.924 | 0.860 | 0.989 | 0.867 |
| 1/Matsuda^D^ | 0.827 | 0.684 | 0.969 | 0.088 |
| HOMA-AD | 0.778 | 0.612 | 0.943 | 0.042 |
| 1/Avignon^D^ | 0.760 | 0.598 | 0.923 | 0.024 |
| 1/Fasting insulin sensitivity index (ISI_0min_)^S^ | 0.760 | 0.596 | 0.924 | 0.023 |
| Homeostatic model assessment (HOMA)-IR^S^ | 0.760 | 0.596 | 0.924 | 0.002^b^ |
| Fasting insulin resistance index (FIRI)^S^ | 0.760 | 0.596 | 0.924 | 0.002^b^ |
| 1/Bennet^S^ | 0.749 | 0.580 | 0.918 | 0.002^b^ |
| 1/HOMA-2-IS^S^ | 0.738 | 0.568 | 0.908 | 0.002^b^ |
| Fasting insulin^S^ | 0.729 | 0.557 | 0.900 | 0.001^b^ |
| 1/Raynaud^S^ | 0.729 | 0.557 | 0.900 | 0.010 |
| 1/McAuley^S^ | 0.729 | 0.557 | 0.900 | 0.011 |
| HOMA-2-IR^S^ | 0.734 | 0.569 | 0.899 | 0.010 |
| 1/Adiponectin | 0.733 | 0.541 | 0.926 | 0.045 |
| 1/Stumvoll without demographics^D^ | 0.687 | 0.578 | 0.795 | p<0.001^b^ |
| Quantitative insulin sensitivity check index (QUICKI)^S^ | 0.657 | 0.473 | 0.841 | p<0.001^b^ |
| Fasting insulin/fasting glucose ratio^S^ | 0.663 | 0.481 | 0.846 | p<0.001^b^ |
| 2h-insulin/2h-glucose ratio^D^ | 0.742 | 0.611 | 0.873 | p<0.001^b^ |
| 1/2h-insulin sensitivity index (ISI_120min_)^D^ | 0.742 | 0.611 | 0.873 | p<0.001^b^ |
| 1/Stumvoll with demographics^D^ | 0.485 | 0.371 | 0.599 | p<0.001^b^ |

**Waist circumference**

**5. Performance in patients with normal waist circumference (men < 94cm & women <80cm) (n=17):**

| Insulin resistance index | AUC | 95% CI | | *P* value^a^ |
| --- | --- | --- | --- | --- |
| 1/Gutt^D^ | 0.867 | 0.659 | 1.000 | - |
| 1/Oral glucose insulin sensitivity index (OGIS)^D^ | 0.933 | 0.789 | 1.000 | 0.172 |
| 1/Matsuda^D^ | 0.867 | 0.616 | 1.000 | 1.000 |
| HOMA-AD | 0.933 | 0.782 | 1.000 | 0.502 |
| 1/Avignon^D^ | 0.933 | 0.782 | 1.000 | 0.514 |
| 1/Fasting insulin sensitivity index (ISI_0min_)^S^ | 0.900 | 0.699 | 1.000 | 0.742 |
| Homeostatic model assessment (HOMA)-IR^S^ | 0.900 | 0.699 | 1.000 | 0.737 |
| Fasting insulin resistance index (FIRI)^S^ | 0.900 | 0.699 | 1.000 | 0.737 |
| 1/Bennet^S^ | 0.900 | 0.699 | 1.000 | 1.000 |
| 1/HOMA-2-IS^S^ | 0.867 | 0.654 | 1.000 | 1.000 |
| Fasting insulin^S^ | 0.833 | 0.581 | 1.000 | 0.662 |
| 1/Raynaud^S^ | 0.833 | 0.581 | 1.000 | 0.789 |
| 1/McAuley^S^ | 0.800 | 0.505 | 1.000 | 0.657 |
| HOMA-2-IR^S^ | 0.733 | 0.384 | 1.000 | 0.419 |
| 1/Adiponectin | 0.900 | 0.724 | 1.000 | 0.780 |
| 1/Stumvoll without demographics^D^ | 0.533 | 0.050 | 1.000 | 0.081 |
| Quantitative insulin sensitivity check index (QUICKI)^S^ | 0.800 | 0.505 | 1.000 | 0.449 |
| Fasting insulin/fasting glucose ratio^S^ | 0.800 | 0.505 | 1.000 | 0.455 |
| 2h-insulin/2h-glucose ratio^D^ | 0.833 | 0.535 | 1.000 | 0.264 |
| 1/2h-insulin sensitivity index (ISI_120min_)^D^ | 0.833 | 0.535 | 1.000 | 0.264 |
| 1/Stumvoll with demographics^D^ | 0.667 | 0.133 | 1.000 | 0.078 |

**6. Performance in patients with large waist circumference (men ≥94cm & women ≥80cm) (n=166):**

| Insulin resistance index | AUC | 95% CI | | *P* value^a^ |
| --- | --- | --- | --- | --- |
| 1/Gutt^D^ | 0.874 | 0.802 | 0.946 | - |
| 1/Oral glucose insulin sensitivity index (OGIS)^D^ | 0.856 | 0.776 | 0.935 | 0.565 |
| 1/Matsuda^D^ | 0.780 | 0.680 | 0.880 | 0.023 |
| HOMA-AD | 0.742 | 0.636 | 0.849 | 0.007 |
| 1/Avignon^D^ | 0.735 | 0.635 | 0.834 | 0.003 |
| 1/Fasting insulin sensitivity index (ISI_0min_)^S^ | 0.754 | 0.656 | 0.852 | 0.007 |
| Homeostatic model assessment (HOMA)-IR^S^ | 0..754 | 0.656 | 0.852 | 0.003 |
| Fasting insulin resistance index (FIRI)^S^ | 0.754 | 0.656 | 0.852 | 0.003 |
| 1/Bennet^S^ | 0.728 | 0.627 | 0.829 | 0.001^b^ |
| 1/HOMA-2-IS^S^ | 0.716 | 0.614 | 0.818 | 0.001^b^ |
| Fasting insulin^S^ | 0.700 | 0.597 | 0.802 | p<0.001^b^ |
| 1/Raynaud^S^ | 0.700 | 0.597 | 0.802 | p<0.001^b^ |
| 1/McAuley^S^ | 0.698 | 0.595 | 0.801 | p<0.001^b^ |
| HOMA-2-IR^S^ | 0.685 | 0.583 | 0.788 | p<0.001^b^ |
| 1/Adiponectin | 0.620 | 0.488 | 0.751 | p<0.001^b^ |
| 1/Stumvoll without demographics^D^ | 0.703 | 0.613 | 0.793 | p<0.001^b^ |
| Quantitative insulin sensitivity check index (QUICKI)^S^ | 0.633 | 0.522 | 0.744 | p<0.001^b^ |
| Fasting insulin/fasting glucose ratio^S^ | 0.634 | 0.526 | 0.742 | p<0.001^b^ |
| 2h-insulin/2h-glucose ratio^D^ | 0.631 | 0.528 | 0.733 | p<0.001^b^ |
| 1/2h-insulin sensitivity index (ISI_120min_)^D^ | 0.631 | 0.528 | 0.733 | p<0.001^b^ |
| 1/Stumvoll with demographics^D^ | 0.519 | 0.426 | 0.611 | p<0.001^b^ |

CI, confidence interval
^a^ *p* value indicates AUC comparison with the reciprocal of Gutt index AUC.

^b^Significant statistical difference (*p*<0.0024).
