# Supplementary material for: Accuracy of insulin resistance indices for metabolic syndrome: a cross-sectional study in adults
Source: Diabetol Metab Syndr. 2018 Aug 20;10:65. doi: 10.1186/s13098-018-0365-y (PMC6102896; doi:10.1186/s13098-018-0365-y)
Supplement: Supplementary file 5 — Additional file 5. Number of metabolic syndrome criteria by median of insulin sensitivity and resistance indices. The tables show a comparison between groups divided by median of selected insulin sensitivity and resistance indices for number of metabolic syndrome criteria. [file 13098_2018_365_MOESM5_ESM.docx]

**Additional file 5 Number of metabolic syndrome criteria by median of insulin sensitivity and resistance indices**

**Insulin sensitivity indices**

| Gutt index | Gutt < 2.754 | Gutt **≥** 2.754 | *P* value^a^ |
| --- | --- | --- | --- |
| Number of participants | 92 | 91 | - |
| Number of metabolic syndrome criteria | 4 (3 - 5) | 3 (2 - 3) | <0.001^b^ |

| OGIS index | OGIS < 344 | OGIS **≥** 344 | *P* value^a^ |
| --- | --- | --- | --- |
| Number of participants | 91 | 92 | - |
| Number of metabolic syndrome criteria | 4 (3 - 5) | 3 (2 - 3.8) | <0.001^b^ |

**Insulin resistance indices**

| HOMA-IR index | HOMA-IR < 2.70 | HOMA-IR **≥** 2.70 | *P* value^a^ |
| --- | --- | --- | --- |
| Number of participants | 91 | 92 | - |
| Number of metabolic syndrome criteria | 3 (2 - 4) | 4 (3 - 5) | <0.001^b^ |

| Fasting insulin (Ins_0min_) | Ins_0min_ < 10.63 | Ins_0min_ **≥** 10.63 | *P* value^a^ |
| --- | --- | --- | --- |
| Number of participants | 91 | 92 | - |
| Number of metabolic syndrome criteria | 3 (2 - 4) | 4 (3 - 5) | <0.001^b^ |

Data are expressed as median (P25–75).

^a^P value for comparisons between two groups was tested by χ^2^ test for categorical variables or Student’s *t*-test for continuous variables.

^b^Significant statistical difference (*p*<0.05).
